# Extensive Pyoderma gangrenosum following breast reduction and abdominoplasty: a challenging case

**DOI:** 10.1080/23320885.2024.2302124

**Published:** 2024-01-23

**Authors:** Vera Obinwanne, Nathalia Hoffmann Guarda, Erinolaoluwa Araoye, Alaina J. James

**Affiliations:** a Western Michigan Homer Stryker School of Medicine, Kalamazoo, Michigan, USA; b Department of Dermatology, University of Pittsburgh Medical Center, Pittsburgh, Pennsylvania, USA; c Federal University of Health Sciences of Porto Alegre (UFCSPA), Porto Alegre, Brazil; d University of Pittsburgh School of Medicine, Pittsburgh, Pennsylvania, USA

**Keywords:** Pyoderma gangrenosum, post-surgical pyoderma gangrenosum, breast reduction surgery, abdominoplasty, mummy make over

## Abstract

Post-surgical pyoderma gangrenosum (P SP G) is a subtype of pyoderma gangrenosum in which non-infectious, painful, inflammatory ulcerative nodules develop in incision sites. Delayed diagnosis and surgical interventions of P SP G often contribute to worsened morbidity. We present a case of a 55-year-old female diagnosed with severe P SP G after breast augmentation and abdominoplasty.

## Introduction

Pyoderma gangrenosum (PG) is a rare neutrophilic dermatosis of unclear etiology postulated to involve dysfunctional neutrophil chemotaxis and immune dysregulation [1, 2]. PG presents as painful inflammatory nodules and rapidly expanding, suppurative ulcers with necrotic centers [1, 3]. The ulcers are typically tender with inflamed rolled borders [4]. As PG and skin infections have a similar clinical appearance, the diagnosis and treatment of PG is often delayed. PG can be induced and worsened by cutaneous trauma, a process known as pathergy, and can also occur spontaneously [3–5]. Post-surgical pyoderma gangrenosum (PSPG) is a subtype of PG in which surgery triggers exuberant neutrophilic inflammation resulting in the development of PG ulcers in surgical incisions [3]. We present a case of PSPG in a woman, one week after cosmetic breast reduction and abdominoplasty.

## Case summary

**Figure 1.**
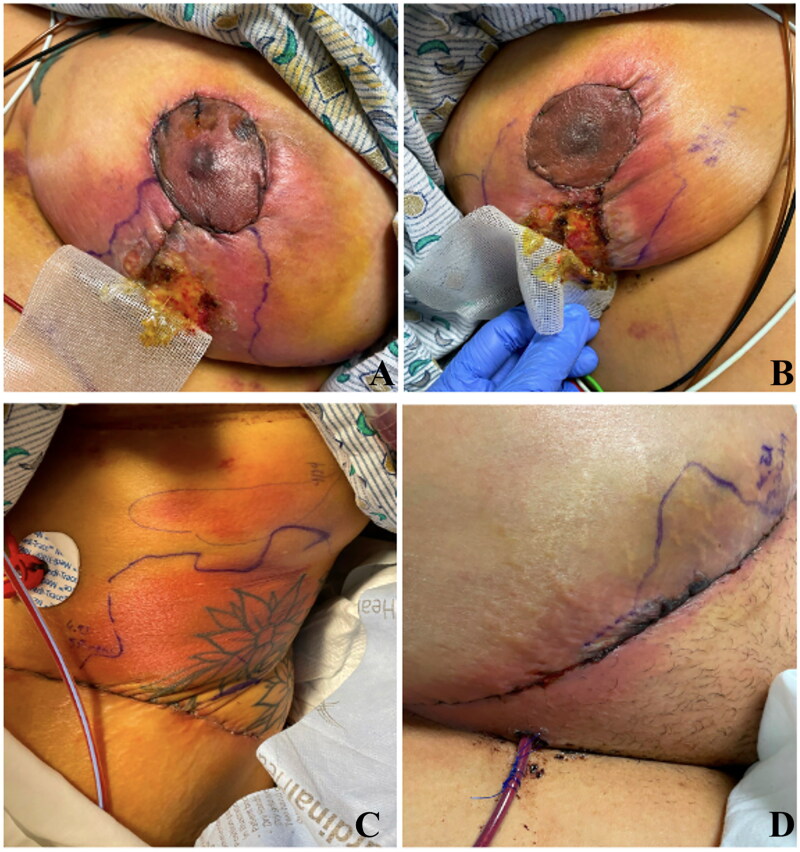
One-week post breast reduction and abdominoplasty: Patient presented with tender pink-red firm patches and nodules, swelling of the skin around the incision sites, wound dehiscence, with overlying crust. A: Right breast incision site B: Left breast incision site C: Abdominal incision site D: Left lateral abdomen incision site.

A 55-year-old female presented to the emergency department with rigors, chills, and worsening breast and lower abdominal pain one week after breast reduction surgery and abdominoplasty. On physical examination, she was febrile (38.3 °C) and had tender pink-red firm nodules and swelling of surgical sites of the breast, abdomen, and flank, with areas of wound dehiscence and overlying serosanguinous crust (Figure 1). Her bloodwork was notable for mild leukocytosis (11.8 × 10^9^/L), elevated CRP (34.6 mg/dL), and mildly elevated liver function tests (AST:140 IU/L, ALT:213 IU/L, AP:335 IU/L). EKG, chest x-ray, and CT of the chest, abdomen, and pelvis were unremarkable. Her past medical history was significant for well-controlled hypertension, pernicious anemia, and remote, uncomplicated hysterectomy and appendectomy.

She was admitted and treated for suspected postoperative cellulitis with vancomycin, linezolid, azithromycin, and cefepime; however, she experienced worsening pain and increasing redness and swelling at the surgical closure sites, with expanding wound dehiscence and ulcerations. Infectious work up including blood cultures, MRSA screen, tissue cultures, and tissue stains of Gram, GMS, PAS, Fite, and AFB were initially negative for microorganisms. After a 10-day course of antibiotics, a repeat superficial wound culture grew Corynebacterium striatum.

**Figure 2.**
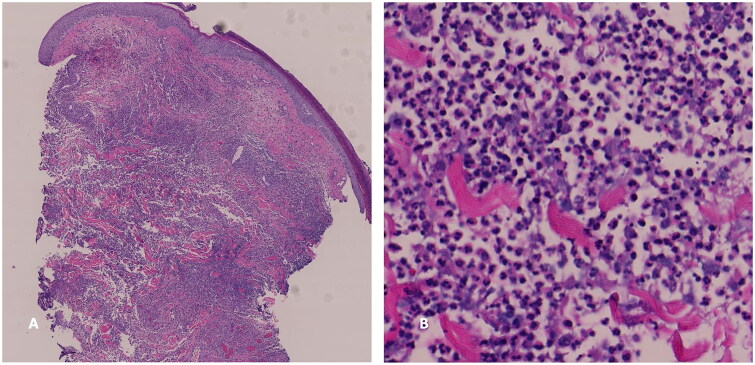
Punch biopsy of ulcer edge. Hematoxylin and eosin stain demonstrating exuberant neutrophilic inflammation in the dermis (A) 20× (B) 200×.

**Figure 3.**
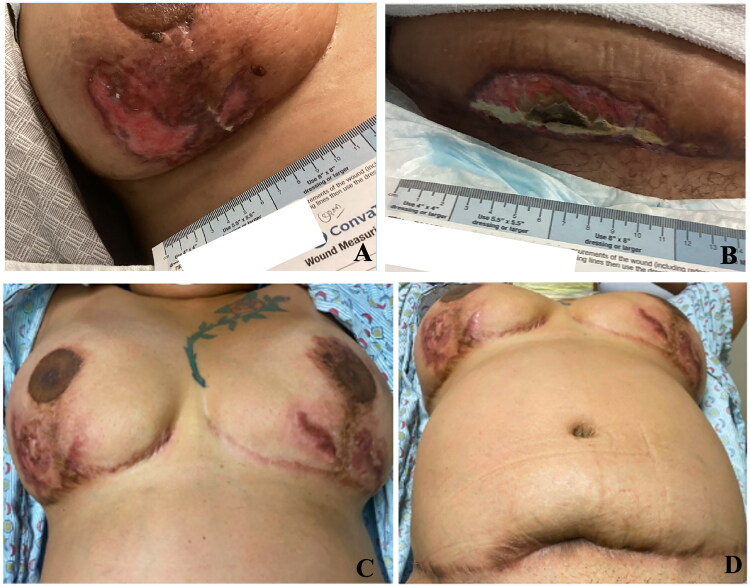
A: Right breast following 3 months of treatment. B: Abdomen after 3 months of treatment. Complete healing with cribriform scar after 8 months of treatment. C: Right and left breast after 8 months of treatment. D: Abdomen after 8 months of treatment.

A multidisciplinary team of infectious disease, plastic surgery, and dermatology health professionals were consulted to discuss management including proposed surgical intervention and debridement. A punch biopsy of the left breast revealed focal ulceration overlying a dense neutrophil-predominant inflammatory infiltrate throughout the dermis, clinicopathologically consistent with pyoderma gangrenosum (Figure 2). She was treated with high-dose prednisone, topical mupirocin, topical hydrocortisone, and wound care with significant improvement. At the 3-week outpatient follow-up visit, she was started on cyclosporine, and prednisone was slowly tapered. Her outpatient rheumatology workup and cancer screening (mammography and colonoscopy) were negative. During the first 3 months of PG treatment, she received pain management and at-home wound care due to limited physical capacity. At her ten-month follow-up visit, she had well-healed scars with decreased pain and improved quality of life (Figure 3). The patient was tapered off cyclosporine and prednisone with no recurrence of PG.

## Discussion

PSPG is a rare condition which can occur in post-surgical incision scars, typically 1 to 6 weeks after surgery [1]. Based on the clinical presentation of painful ulcers, ecthyma gangrenosum, atypical mycobacteria, malignant cancers, drug induced bromoderma, pemphigus vegetans, and granulomatous vasculitis are considered on the differential diagnosis.

**Figure 4.**
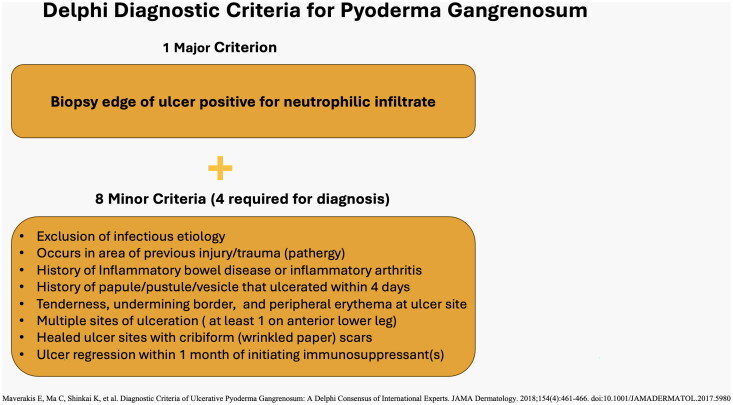
Delphi diagnostic criteria for ulcerative Pyoderma gangrenosum [6].

Due to the broad differential diagnosis, the diagnosis of PG is often delayed. Time to diagnosis for PSPG can be up to 3 years [1]. PG can be idiopathic but is commonly associated with inflammatory bowel disease, rheumatoid arthritis, and hematologic malignancies. In contrast, PSPG is typically not associated with systemic disease [3, 5]. PSPG has anatomical propensity for the breast and abdomen, therefore surgical procedures in these areas may confer a higher likelihood of developing the condition [5]. Surgeons should suspect PSPG in patients presenting with painful, rapidly expanding necrotic ulcers days to weeks after surgical procedures involving the breast and abdomen. While historically considered a diagnosis of exclusion, new diagnostic criteria for the ulcerative variant of the disease have been proposed based on expert consensus using the Delphi technique. The proposal includes 1 major criterion and 8 minor criteria that when combined with high clinical suspicion, may have utility in accelerating diagnosis of PSPG (Figure 4) [6].

Resolution of PSPG can take months to achieve [1]. Systemic corticosteroids and cyclosporine are often first-line PSPG treatments and can be used in combination or as monotherapy. Anti-interleukin therapy and tumor necrosis factor inhibitors can be used adjunctively or as second line therapy in cases of severe PSPG. With the concern for a possible invasive infection, surgical teams may consider debridement as a treatment option for this condition; however, PG is a condition which often is exacerbated by trauma. By debriding PG lesions, a person may experience increasing size of the lesions, worsening pain, and subsequently worse cosmetic outcomes. Health professionals should maintain a high level of clinical suspicion for PG to avoid interventions such as debridement which can cause further progression and increase the morbidity of PG wounds [1, 3].

## Conclusion

PG is a rare but serious and severe condition that can be triggered by surgery. Delayed diagnosis and surgical interventions can worsen pain and disease progression. We highlight the importance of early recognition, prompt treatment, and avoidance of surgical debridement in the management of PSPG.

## References

[1] Ehrl DC, Heidekrueger PI, Broer PN. Pyoderma gangrenosum after breast surgery: a systematic review. J Plast Reconstr Aesthet Surg. 2018;71(7):1–4. doi: 10.1016/J.BJPS.2018.03.013. 29748073

[2] Maverakis E, Marzano AV, Le ST, et al. Pyoderma gangrenosum. Nat Rev Dis Primers. 2020;6(1):81. doi: 10.1038/S41572-020-0213-X. 33033263

[3] Mella JR, Maselli AM, Guo L. A deceptive diagnosis: pyoderma gangrenosum after breast surgery-a case series and literature review. Ann Plast Surg. 2019;83(4S Suppl 1):S21–S30. doi: 10.1097/SAP.0000000000002101. 31513063

[4] Zuo KJ, Fung E, Tredget EE, et al. A systematic review of post-surgical pyoderma gangrenosum: identification of risk factors and proposed management strategy. J Plast Reconstr Aesthet Surg. 2015;68(3):295–303. doi: 10.1016/J.BJPS.2014.12.036. 25589459

[5] Tolkachjov SN, Fahy AS, Wetter DA, et al. Postoperative pyoderma gangrenosum (PG): the Mayo clinic experience of 20 years from 1994 through 2014. J Am Acad Dermatol. 2015;73(4):615–622. doi: 10.1016/J.JAAD.2015.06.054. 26209218

[6] Maverakis E, Ma C, Shinkai K, et al. Diagnostic criteria of ulcerative pyoderma gangrenosum: a delphi consensus of international experts. JAMA Dermatol. 2018;154(4):461–466. doi: 10.1001/jamadermatol.2017.5980. 29450466

